# The boot camp program for lumbar spinal stenosis: a protocol for a randomized controlled trial

**DOI:** 10.1186/s12998-016-0106-y

**Published:** 2016-07-18

**Authors:** Carlo Ammendolia, Pierre Côté, Y. Raja Rampersaud, Danielle Southerst, Brian Budgell, Claire Bombardier, Gillian Hawker

**Affiliations:** Institute of Health Policy, Management and Evaluation, University of Toronto, Toronto, Canada; Rebecca MacDonald Centre for Arthritis & Autoimmune Disease, Mount Sinai Hospital, Toronto, Canada; Dalla Lana School of Public Health, University of Toronto, Toronto, Canada; University of Ontario Institute of Technology, Ontario, Canada; Department of Orthopedics, Toronto Western Hospital, University Health Network, Toronto, Canada; Canadian Memorial Chiropractic College, Toronto, Ontario Canada; Division of Rheumatology, Department of Medicine, University of Toronto, Toronto, Canada; Department of Medicine, University of Toronto, Toronto, Ontario Canada

**Keywords:** Lumbar spinal stenosis, Neurogenic claudication, Self-management, Walking, Randomized controlled trial, Boot camp program, Multi-modal treatment, Non-surgical

## Abstract

**Background:**

Lumbar spinal stenosis (LSS) causing neurogenic claudication is a leading cause of pain, disability and loss of independence in older adults. The prevalence of lumbar spinal stenosis is growing rapidly due to an aging population. The dominant limitation in LSS is walking ability. Postural, physical and psychosocial factors can impact symptoms and functional ability. LSS is the most common reason for spine surgery in older adults yet the vast majority of people with LSS receive non-surgical treatment. What constitutes effective non-surgical treatment is unknown. The purpose of this study is to evaluate the effectiveness of a multi-modal and self-management training program, known as the Boot Camp Program for LSS aimed at improving walking ability and other relevant patient-centred outcomes.

**Methods:**

We will use a pragmatic two-arm randomized controlled single blinded (assessor) study design. Eligible and consenting participants will be randomized to receive from licensed chiropractors either a 6-week (twice weekly) self-management training program (manual therapy, education, home exercises) with an instructional workbook and video and a pedometer or a single instructional session with an instructional workbook and video and pedometer. The main outcome measure will be the self-paced walking test measured at 6 months. We will also assess outcomes at 8 weeks and 3 and 12 months.

**Discussion:**

Symptoms and functional limitations in LSS are variable and influenced by changes in spinal alignment. Physical and psychological factors result in chronic disability for patients with LSS. The Boot Camp Program is a 6-week self-management training program aimed at the multi-faceted aspects of LSS and trains individuals to use self-management strategies. The goal is to provide life-long self-management strategies that maximize walking and overall functional abilities and quality of life.

**Trial registration:**

ClinicalTrials.gov ID: NCT02592642.

## Background

Lumbar spinal stenosis (LSS) refers to an anatomical narrowing of the central and/or lateral spinal canals [1]. It is usually caused by age related degenerative changes in the spine including intervertebral disc thinning, facet joint thickening and in-folding of the ligamentum flavum [2]. These changes contribute to a decrease in cross-sectional area of the spinal canal that can lead to compression and diminished blood flow to the spinal nerves [3, 4]. The clinical syndrome caused by LSS is known as neurogenic claudication. This syndrome is characterized by bilateral or unilateral buttock, lower extremity pain, heaviness, numbness, tingling or weakness, precipitated by walking and standing and [5] relieved by sitting and bending forward [2, 6]. Neurogenic claudication due to LSS is a leading cause of pain, disability and loss of independence in people over the age of 65 [7]. Limited walking ability is the dominant functional impairment caused by LSS [2]. Those afflicted have greater walking limitations than individuals with knee or hip osteoarthritis [8] and greater functional limitations than those with congestive heart failure, chronic obstructive lung disease or systemic lupus erythematosus [7]. Inability to walk among individuals with LSS leads to a sedentary lifestyle and a progressive decline in health status [9, 10]. Furthermore, symptomatic LSS is also associated with increased levels of depression; anxiety and hopelessness that can further perpetuate disability [11–14].

The prevalence and economic burden of LSS is growing exponentially due to the aging population. In Japan where 25 % of the population is over the age of 65, about 12 million people suffer from symptomatic LSS [15]. A similar epidemic is expected in the US by the year 2030 when an estimated 73 million people will be over the age of 65 [16] of which 30 % are projected to have symptomatic LSS [17]. LSS is the most common reason for spine surgery in individuals over the age of 65 [18], however only very few patients receive surgery [19]. The vast majority of individuals with LSS receive non-surgical care. However, what constitutes effective non-surgical care is unknown [20–23].

We have designed and implemented a training program for LSS known as the Boot Camp Program for LSS [24]. This program aims to address the multi-faceted aspects of LSS using a multi-modal non-surgical approach with a focus on self-management and the goals of improved walking ability, overall functional status and quality of life. The program considers the dynamic nature of LSS where symptoms change relative to posture. Reduction of the lumbar lordosis while standing, walking and sitting reduces epidural pressure and improves blood flows to spinal nerves [3, 11]. Enabling patients to introduce inter-segmental lumbar spine flexion (reducing the lumbar lordosis) using anterior pelvic tilt may reduce symptoms and improve walking ability [25–27]. Individuals with LSS tend to be deconditioned due to inability to walk and consequential sedentary lifestyle and may not have the necessary core strength and lumbar flexibility to accomplish this body realignment. They can also have reduced lower extremity strength due to combined nerve root compression and disuse. Specific flexibility, aerobic and core and lower extremity strengthening exercises, manual therapy and postural instruction may overcome these deficiencies.

The program also considers potential psychosocial consequences of LSS and incorporates a cognitive behavioral approach [28, 29]. This approach aims to improve coping and problem solving, build self-efficacy, provide feedback and develop realistic treatment and functional goals. Since LSS is a chronic and often-progressive condition [30] the program emphasizes incorporating learned physical and psychosocial self-management strategies for life. Patients receive an instructional workbook and video as part of the training program. The workbook and video provide education on self-management strategies and instruction on how to perform all the specific exercises and body realignment techniques. The workbook incorporates a schedule outlining the intensity and frequency of each exercise with goals tailored to each patient. The training program also includes a pedometer that provides weekly feedback for both patients and practitioners on walking ability.

In a previous retrospective study we evaluated 49 consecutive patients who completed the Boot Camp Program and assessed the difference in self-report outcomes selected a priori [24]. Outcome measures included the Oswestry Low Back Pain Disability Index, the walking score of the Oswestry Low Back Pain Disability Index, the three subscales of the Zurich Swiss Spinal Questionnaire, and the Numeric Rating Scale for leg and back pain. Following the 6-week intervention there were both statistically and clinically important improvements in all outcomes from baseline. This was a before and after study without a control group and therefore provided low quality preliminary evidence on the effectiveness of the program.

The purpose of this study is to assess the effectiveness of the Boot Camp Program in improving outcomes using a randomized controlled trial (RCT) design. Our main objective is to compare the effectiveness of a comprehensive 6-week self-management training program that includes a patient instructional workbook, video and pedometer to a single training session with provision of a patient workbook, video and pedometer. We hypothesize that the self-management training program will be more effective in improving walking capacity and functional outcomes than a single training session.

## Methods

### Design

**Fig. 1:**
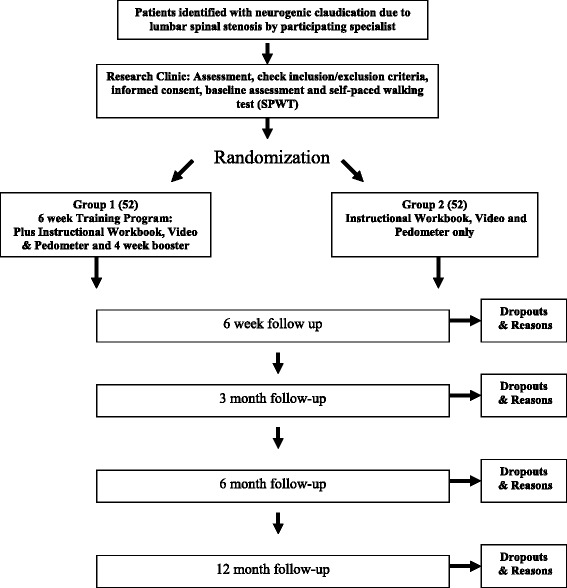
Study Flow Process of f assessment

We will conduct a pragmatic two arm single blinded (assessor) RCT (Fig. 1).

### Source population

Eligible participants are individuals who consult with specialists (orthopedists, neurosurgeons, rheumatologists, neurologists or physiatrists), family physicians or chiropractors for symptoms suggestive of neurogenic claudication at one of seven hospitals and several community clinics in Toronto, Canada and the surrounding area.

### Recruitment

Participating physicians and chiropractors will identify potential participants using an eligibility checklist (Table 1). Eligible potential participants will be given a pamphlet outlining information concerning the study and providing contact information for the trial coordinator. Interested and potentially eligible participants will be asked to contact the trial coordinator directly. Pamphlets will also be available in patient waiting rooms of participating hospital clinics and community clinics. Similar information will be advertised in local newspapers to enhance recruitment. The trial coordinator will provide details about the study and answer questions by phone. The coordinator will confirm eligibility including age, duration of symptoms and self-report walking ability. Walking ability will be assessed by the participants’ response to the question *“are you able to walk continuously for 30 min without the use of aids or without stopping to rest or stoop forward to alleviate your symptoms?*” Self-reported walking ability has been shown to be highly correlated (*r* = 0.80) to the self-paced walking test (SPWT) [31]. The SPWT is a validated objective measure of walking ability in LSS [31]. Only interested participants who respond “NO” and meet the other inclusion/exclusion criteria will be given an appointment for an intake assessment at the study site (Mount Sinai Hospital in Toronto Canada).

**Table 1:** Inclusion and exclusion criteria

Inclusion criteria	
1. Age greater or equal to 50 years	
2. Clinical symptoms of back and/or radiating lower limb or buttock pain; fatigue or loss of sensation in the lower limbs aggravated by walking and/or standing and relieved by sitting.	
3. Intermittent or persistent pain without progressive neurological dysfunction	
4. Duration of symptoms and signs for more than 3 months	
5. Imaging confirmed spinal canal narrowing using MRI, CT scan, myelography or ultrasound	
6. Clinical signs and symptoms corresponding to segmental level of narrowing identified by imaging	
7. Patients with degenerative spondylolisthesis are included	
8. Not considered to be a surgical candidate (in the next 12 months) or patient unwilling to have surgery	
9. Able to perform mild-moderate exercise	
10. Able to walk without assistive devices for at least 20 m and less than 30 min continuously	
11. Able to give written informed consent and complete interviews and questionnaires in English.	
Exclusion criteria	
1. Severe degenerative stenosis with intractable pain and progressive neurological dysfunction	
2. Lumbar spinal stenosis not caused by degeneration	
3. Lumbar herniated disc diagnosed during the last 12 months	
4. Previous back surgery for lumbar spinal stenosis or instability	
5. Underlying spinal disorder such as ankylosing spondylitis, neoplasm, infection or metabolic disease	
6. Intermittent claudication due to vascular disease	
7. Severe osteoarthrosis or arthritis of lower extremities causing limited walking ability	
8. Neurologic disease causing impaired function of the lower limbs, including diabetes	
9. Psychiatric disorders and/or cognitively impaired	

### Inclusion and exclusion criteria

At the intake assessment, a licensed practitioner will assess potential participants. The assessment will confirm eligibility (Table 1) and will include a history, physical examination and a review of imaging results provided by the referring specialist. Eligible and willing participants will be asked to provide informed written consent. Consenting participants will be asked to complete a baseline questionnaire, a short physical performance battery (SPPB) [32] and perform a 30-min SPWT [31].

### Randomization to treatment groups

Randomization will take place following the completion of the consent process, baseline questionnaire, SPPB and the SPWT (Table 2). All participants will be randomly allocated to Group 1, the self-management training program with instructional workbook, video and pedometer or Group 2, a single instructional session and the instructional workbook, video and pedometer (Fig. 1). The randomization sequence will be prepared by the study biostatistician ahead of recruitment using a computerized random numbers table [NQuery Advisor® 7.0 [33]]. The biostatistician will provide the trial coordinator with sequentially numbered sealed opaque envelopes containing the allocation for Group 1 or 2. The study biostatistician will not be involved in the selection, treatment or follow-up of participants.

**Table 2:** Measures collected at baseline and follow-up periods

Measures	Baseline	6 Weeks	3 Months	6 Months	12 Months
Socio-demographic characteristics	x				
Duration of symptoms (back or leg)	x				
Dominant pain (back or leg)	x				
Co-Morbidity Disease Index	x				
Self Paced Walking Test	x	x	x	x	x
Zurich Claudication Questionnaire (ZCQ) Symptom and Functional scales	x	x	x	x	x
Oswestry Disability index (ODI) and ODI walk	x	x	x	x	x
Numerical rating scale for back pain	x	x	x	x	x
Numerical rating scale for leg pain	x	x	x	x	x
36-item short-form health survey (V2)	x	x	x	x	x
Center for Epidemiological Studies-Depression Scale (CES-D)	x	x	x	x	x
Short Physical Performance Battery (SPPB)	x	x	x	x	x
Falls Efficacy Scale (FES)	x	x	x	x	x
Co-interventions and compliance		x	x	x	x

### Interventions and controls

Participants will be scheduled to begin either the self-management training program (Group 1) or the single instructional session (Group 2) based on their random assignment.

### Group 1: self management training program with instructional workbook, video and pedometer

Participants randomly assigned to Group 1 will receive the self management-training program with instructional workbook, video and pedometer at the study centre. All instruction and treatments will be provided by licensed chiropractors. Participants will be scheduled for twelve, 15-min sessions over a 6-week period (two treatment sessions per week). Four weeks following the 6-week treatment period a follow-up treatment (booster) session will be scheduled. Participants will be scheduled as regular patients at the Chiropractic Spine Clinic and Spinal Stenosis Program at Mount Sinai Hospital. All appointments will be confirmed 24 h in advance via telephone or e-mail as preferred by the participant as per usual treatment protocol. The intent is to provide participants with treatment that simulates real practice in terms of scheduling, time spent and content of treatment.

The Group 1 participants will receive the following:

- **Education:** Participants will receive instruction on self-management strategies using a cognitive behavioural approach [29]. Treating practitioners will provide information on the causes of pain and disability due to LSS, its natural history and prognosis. They will receive instruction on how to manage symptoms and maintain daily routines using problem solving, pacing, relaxation and body positioning [29, 34]. Reassurance, positive re-enforcement, goal setting and graded activity will be used to reduce pain related fear and improve self-efficacy [29, 35] and to improve function [36]. Participants will be instructed on how to reduce the lumbar lordosis when standing and walking using body repositioning techniques (the pelvic tilt).
- **Exercises:** Participants will receive instruction on muscle stretching, strengthening and conditioning exercises directed at improving overall back and lower extremity fitness and facilitating lumbar flexion [26, 37]. Tight muscles that promote lumbar extension (quadriceps, hip flexors, iliopsoas and erector spinae) will be progressively stretched and muscles that promote and control lumbar flexion will be strengthened (upper and lower abdominals). Exercise instruction will be provided and reviewed at each session and will be part of a progressive structured home exercise program. A graduated walking and/or stationary cycling program to improve lower extremity conditioning and overall fitness will be part of the home exercise program [38]. A written exercise and conditioning program schedule will be provided to participants outlining the type, frequency and intensity of the exercises to be performed. The exercises are to be performed twice per day at home with the number, intensity and frequency of each exercise increasing each week for a period of 6 weeks.
- **Manual Therapy:** The aim of the manual therapy will be to improve the flexibility of the lumbar spine and to facilitate lumbar inter-segmental flexion. At each session, manual therapy will be directed to the lumbar and thoracic spine, pelvis and lower extremities. Specific techniques will include low amplitude high velocity manipulation [26], joint, soft tissue, and neural mobilization [26, 39–41], lumbar flexion-distraction [27, 42], and manual muscle stretching [25]. The specific combination of manual therapy techniques used will be at the discretion of the treating practitioner based on identified underlying functional impairments.

Education, exercise instruction and manual therapy will be provided at each session and tailored to the needs of the participant by the treating chiropractor. An instructional workbook and video will be provided to all participants. This workbook and video provide education and a step-by-step guide on how to perform all the exercises and body realignment techniques, and are aimed at reinforcing the instructions received during the training sessions. The workbook also includes a diary to record exercise and self-management activities during the study period. Participants will also be provided a pedometer (Pedusa PE-771, Pedometers USA) with instruction and asked to record once per week the maximum number of continuous walking steps and time (minutes) to stop walking due to neurogenic symptoms.

### Group 2: single instructional session with instructional workbook, video and pedometer

Participants randomized to this group will receive the instructional workbook, video and pedometer provided and explained in a single 15–30 min session with a experienced licensed chiropractor not involved in the provision of the self-management training program. The emphasis on the instructional session is on reviewing the material in the workbook and the structure of the 6 week exercise program. No manual therapy will be provided during the session.

### Data collection and follow-up

Table 2 summarizes the data to be collected during the trial. We will follow-up with participants at 6 weeks, and 3, 6 and 12 months following randomization. A trained blinded assessor will conduct all follow-up assessments at the study site. Should patients be unable to attend a follow-up in person, all self-report measures will be administered via telephone. All assessors will receive training in order to standardize assessments and minimize inter- and intra-assessor variability. The assessors will be blinded to the treatment allocation and will be responsible for all follow-up assessment per assigned participant. The following outcomes will be measured at each follow-up.

### Outcomes

#### Primary outcome

##### Objective walking capacity

Walking capacity will be assessed using the SPWT. The test requires subjects to walk on a level surface without support at their own pace until forced to stop due to symptoms of LSS or a time limit of 30 min [43]. Test termination will be defined as a complete stop of 3 s. A blinded assessor will follow one metre behind the subject, without conversing, with a distance instrument (Lufkin Pro-Series Model PSMW38), and stopwatch. Distance walked and time to test termination will be recorded. The SPWT is considered the gold standard with high validity for assessing walking capacity in this population since it directly observes walking ability under conditions representative of a real world setting [31, 44]. It has high test-retest reliability (ICC = 0.98) [43]. The minimal clinically important difference (MCID) in walking distance in this population is unknown.

#### Secondary outcomes

##### Physical function

This will be measured using the physical performance scale of the Zurich Claudication Questionnaire (ZCQ) also known as the Swiss Spinal Stenosis Scale. The ZCQ is a validated condition-specific measure consisting of three scales; a physical performance scale, a symptom severity scale and a patient satisfaction scale [45, 46]. The physical performance scale consists of five questions related to walking ability. The mean un-weighted score will be calculated. The scale has a high internal consistency with a Cronbach’s coefficient of 0.91, a test-retest reliability correlation coefficient of 0.82 and a responsiveness of 1.07 using the standardized mean [45, 46]. The MCID has been estimated to be 0.5 [45].

##### Symptom severity

This will be measured using the symptom severity scale of ZCQ. The symptom scale consists of seven questions pertaining to overall severity of pain, pain frequency, back pain and, pain in the leg, numbness, weakness and balance disturbance. The scale has a high internal consistency with a Cronbach’s coefficient of 0.87, a test-retest reliability correlation coefficient of 0.92 and a responsiveness of 0.86 using the standardized mean [45, 46]. The MCID is estimated to be 0.5 [45].

##### Functional disability

Functional disability will be measured by the Oswestry Disability Index (ODI) [47]. The ODI is a reliable and validated measure of back-related disability where 0 represents no disability and 100 represent the worse possible disability. We will also record separately the score of the walking section (ODI walk) of the ODI. The ODI walk score has been shown to be highly correlated to objective walking distance (*r* = 0.83). The MCID for the ODI is 8–12 percentage points [48].

##### Leg and back pain intensity while walking

Leg and back pain intensity while walking will be independently measured at baseline and at each follow-up with the 11-point numerical rating scale (NRS). The NRS is a global measure of pain intensity anchored by two extremes of pain intensity ranging from 0 (referring to “No pain”) to 10 (referring to “Pain as bad as it could be”). The NRS has good short-term test-retest reliability with correlation coefficients ranging from 0.95 to 0.99 when re-administered within 24 h [49]. The NRS has good construct validity and can distinguish between various levels of pain in subjects with chronic post-operative pain [49, 50].

##### Health-related quality of life (H-RQoL)

We will use the Medical Outcomes Study Short-Form Health Survey version two (SF-36) to measure health-related quality of life. The SF-36 has 36 items that measure the H-RQoL of a subject. Two summary scores can be computed: the physical component score and the mental component score. The questionnaire has been shown to have excellent reliability demonstrated with internal consistency and test-retest methods. The SF-36 is a valid and reliable measure for clinical and general populations with a reported intra-class correlation coefficient of 0.85 [51].

##### Co-morbidity scale

We will use the validated and reliable 18-item Co-Morbidity Disease Index that has an emphasis on functional activity [52].

##### Depressive symptomatology

Depressive symptomatology in the previous week will be measured with the Center for Epidemiological Studies-Depression Scale (CES-D). The CES-D is a widely used 20-item self-report scale designed to measure current level of depressive symptomatology in population-based epidemiologic research [53]. It has good test-retest reliability and internal consistency and possesses good factorial and discriminate validity [54]. The CES-D is scored from 0 to 60 with higher scores indicating greater depressive symptomatology [53].

##### Lower extremity function and balance

The Short Physical Performance Battery (SPPB) will be used as an objective assessment of lower extremity function and balance [32]. The SPPB is a collection of timed physical tests including standing balance (tandem, semi-tandem, side by side), 4-m walk and repeated sit-to-stand from a chair. The examination requires 10–15 min to complete on average. The overall score on the SPPB is strongly correlated with current self-reported disability highly predictive of future disability among community dwelling older persons [32]. The individual physical tests making up the SPPB have high inter-rater reliability (kappa = 0.80–1.0) and summary scales have been shown to have good to excellent test-retest reliability (kappa = 0.99) [32, 55]. The internal consistency of the summary scale is adequate (Crochbach’s alpha = 0.76) [56].

##### Fear of falling

Fear of falling is a significant predictor of future falls and can be assessed using the Falls Efficacy Scale (FES). The FES assesses patients’ perception of balance and stability during usual activities of daily living [57, 58]. The test-retest reliability is adequate within the geriatric population (*r* = 0.71) and the instrument has excellent internal consistency (Cronbach’s alpha = 0.91) [57]. Furthermore, scores on the FES have been shown to be moderately to highly correlated with other measures of balance confidence (*r* = 0.55–0.86) [59].

##### Co-interventions

We will measure co-interventions by asking participants to self-report the type and frequency of consultations with other health care providers and the type of interventions received beyond those provided in the trial. This would include physiotherapy, chiropractic, massage therapy, acupuncture, epidural injections and surgery. We will also ask participants if they have used medications and the frequency of use for their back and/or leg symptoms.

##### Compliance

At each treatment visit and each follow-up assessment, we will ask participants about their compliance with their self-management programs (Groups 1 and 2). We will ask how often they are performing their exercises, body alignment techniques and self-management strategies. We will also assess compliance from the weekly exercise diary located in the instructional workbook.

### Statistical Issues

#### Sample size

For the primary RCT we have estimated the sample size for the primary outcome of objective walking capacity based on an estimate of the difference in the proportion of participants who achieve a MCID in walking distance. Since the MCID for the SPWT is unknown we will estimate it to be an improvement in walking distance from baseline of 30 % or more. We estimate a total of 30 % of participants will achieve the estimated MCID in Group 2 and 60 % in Group 1. Based on an estimate of 30 % difference in proportions, a power of 0.8, an alpha of 0.05 and an estimated drop-out rate of 20 %, a minimum of 52 participants per group is estimated to be required to achieve significance using a two-tailed *t*-test for two independent proportions [60]. Our primary end point will be the 6-month follow-up.

#### Statistical analysis

Baseline status of treatment groups will be compared using two-tailed independent samples t tests, Chi squared tests of independence, and Mann-Whitney U tests as indicated. Our analyses will be based on the “intention to treat” principle. Data will contain repeated measurements of the main response variables.

We will analyze the primary outcome (SPWT) by calculating the differences in proportions meeting the MCID using Pearson Chi Squared test with 95 % confidence intervals. To control for potential confounding (sex, education, perceived health status, dominant leg or back pain, and hospital), logistic regression models and generalized estimation equation (GEE) methods will be used [5]. These models will control for baseline differences not balanced by randomization. Dichotomous secondary outcomes will be analyzed similarly.

For continuous secondary outcomes we will first compute the group-specific mean, standard deviation and median at each follow-up interval. Second, we will build ordinary least-square (OLS) models using generalized estimating equation to account for the autocorrelation present in the outcomes [5]. Third, we will test whether the group effects are constant throughout the follow-up periods [5]. Fourth, we will test whether imbalances in the distribution of the baseline covariates confound the group effects. Covariates added to the crude linear model that change any of group regression coefficients by 10 % percent or more will be retained as confounders in the adjusted models [61]. The group effects will be reported as the mean differences and 95 % for each follow-up interval.

### Protection of human subjects and assessment of safety

#### Protection of human subjects

The Mount Sinai Hospital (MSH) Research Ethics Board has approved the study protocol (certificate number 14-0020).

#### Adverse events

We will measure the presence of adverse events that may be associated with each of the interventions. This will take place following each visit for participants enrolled in Group 1. For participants enrolled in Group 1 and 2, adverse events will be assessed at each follow-up visit. We will define adverse events as an unintended sign or symptom of the intervention. These include: significant increase in back and/or lower extremity pain, numbness, tingling, tiredness or claudication and cauda equine syndrome. We will compute the incidence (95 % CI) of each adverse event listed above. The cumulative number of visits will be used as the denominator. Any adverse event that is life threatening or associated with significant disability will be reported to the Mount Sinai Hospital Ethics Review Board.

## Discussion

We selected a pragmatic RCT design for our study to reflect a more real world clinical setting. LSS is a varied and multi-faceted condition and in practice, the approach is to tailor care to the patient’s individual needs and tolerances. Participants randomized to Group 1 will receive the standard approach provided at the Spinal Stenosis Clinic at Mount Sinai Hospital; however tailoring will be required with respect to the intensity and type of exercise and manual therapy techniques used, not unlike usual practice. We selected a RCT design because it is the study design of choice when comparing the effectiveness of interventions.

We considered the SPWT to be the appropriate primary outcome measure to address our primary aim. The SPWT is the current gold standard for measuring objective walking capacity in LSS as it assesses walking ability in a real life setting [43]. We also included the functional scale of the ZCQ which is a self-report measure of walking ability that is highly correlated (*r* = 0.80) to the SPWT [31]. The SPWT is logistically more challenging to perform because of the need of a large walking area and added personnel, time and expense of conducting the test, and added time, expense and inconvenience for participants travelling to the study site for follow-ups. This may result in a higher dropout rate for follow-up assessments. To mitigate this possibility of non-compliance to follow-up SPWTs we included the self-report functional scale of the ZCQ that can easily be assessed by phone. Moreover, we can compare our findings to other studies using this self-report outcome measure.

At our centre we have two long and wide hallways connected by two shorter hallways forming a large rectangular area (140 m long) where participants easily can perform the SPWT.

However, the SPWT has a ceiling effect in that the test measures distance traveled for a maximum of a 30-min period. For this reason we will only include participants who are more severely impaired and cannot complete the SPWT during the baseline assessment.

We have included a 1-month booster session following the 6-week training program. This emulates our current practice protocol at our Spinal Stenosis Clinic at Mount Sinai Hospital. The rationale being that LSS is a chronic condition and patients may benefit from periodic monitoring of their self-management skills and abilities. Periodic reassurance and positive reinforcement may also be of benefit especially among patients with underlying psychosocial barriers such as poor coping skills that accentuate functional limitations. Periodic monitoring and coaching may help to improve longer-term outcomes.

A leading cause of failure of RCTs is the lack of enrollment of sufficient number of participants into the study. We have enlisted a large referral source for potential participants. LSS is a very common condition seen by neurosurgeons and orthopedic spine surgeons, rheumatologists, physiatrists, family physicians and chiropractors. If our enrollment is deemed slow, we will expand our referral source to the University of Toronto vascular surgeons who also see a large number of patients with neurogenic claudication caused by LSS. We will also advertise in local newspapers and seniors publications. In addition, we will distribute pamphlets to be placed in-patient waiting areas at participating hospital clinics and in community clinics. Compliance to follow-ups may be problematic and we plan to provide incentives in the form of covering transportation costs up to $25 per day.

We anticipate that assessor blinding (to assigned intervention) during follow-up assessments will be challenging. We will implement strict rules preventing the communication, regarding participant allocation among participants, the research coordinator and blinded assessors.

Study design limitations include the lack of blinding of both practitioners and patients and this can introduce bias. The multi-modal and pragmatic nature of the design prohibits the determination of the component(s) of the intervention that may be responsible for potential improvements. There is potential for participants to receive other interventions during the intervention and between follow-up periods, which may impact the results. We will compare co-interventions receive between group and comment on the potential impact they may have on the results.

There is a high risk of falls in the LSS population and we need to ensure safety of participants while performing the SPWT. We will train assessors accompanying participants during the SPWT and practice procedures to reduce the risk of falls during the SPWT.

Participants randomized to the single instructional session may not follow the provided instructions, or may perform the exercises incorrectly leading to potential for injury. We will incorporate a safety monitoring protocol and provide participants the opportunity to contact us in an effort to reduce the risk of harm to participants randomized to this intervention.

LSS is a chronic arthritic condition whose prevalence, personal and economic burden is growing exponentially due to the aging population. The vast majority of individuals with LSS receive non-surgical care however, what constitutes effective non-surgical care is unknown. A multi-modal approach with a focus on self-management strategies as outlined in this proposal may be a practical and effective means to improve walking ability, functional status and quality of life in this population.
